# Prolyl-hydroxylase inhibition induces SDF-1 associated with increased CXCR4+/CD11b+ subpopulations and cardiac repair

**DOI:** 10.1007/s00109-017-1543-3

**Published:** 2017-05-26

**Authors:** Santhosh Kumar Ghadge, Moritz Messner, Thi Van Pham, Maximilian Doppelhammer, Andreas Petry, Agnes Görlach, Britta Husse, Wolfgang-Michael Franz, Marc-Michael Zaruba

**Affiliations:** 1 Department of Internal Medicine III, Cardiology and Angiology, Medical University Innsbruck, Innsbruck, Austria; 2 Klinikum Grosshadern, Medical Department I, Ludwig Maximilians University, Munich, Germany; 3 Department of Pediatric Cardiology and Congenital Heart Disease, Experimental Pediatric Cardiology. German Heart Center Munich, TU Munich, Munich, Germany

**Keywords:** SDF-1, CXCR4, CD11b, Prolyl-hydroxylases inhibitors, Myocardial ischemia, HIF-1α

## Abstract

**Abstract:**

SDF-1/CXCR4 activation facilitates myocardial repair. Therefore, we aimed to activate the HIF-1α target genes SDF-1 and CXCR4 by dimethyloxalylglycine (DMOG)-induced prolyl-hydroxylase (PH) inhibition to augment CXCR4+ cell recruitment and myocardial repair. SDF-1 and CXCR4 expression was analyzed under normoxia and ischemia ± DMOG utilizing SDF-1-EGFP and CXCR4-EGFP reporter mice. In bone marrow and heart, CXCR4-EGFP was predominantly expressed in CD45+/CD11b+ leukocytes which significantly increased after myocardial ischemia. PH inhibition with 500 μM DMOG induced upregulation of SDF-1 mRNA in human microvascular endothelial cells (HMEC-1) and aortic vascular smooth muscle cells (HAVSMC). CXCR4 was highly elevated in HMEC-1 but almost no detectable in HAVSMC. In vivo, systemic administration of the PH inhibitor DMOG without pretreatment upregulated nuclear HIF-1α and SDF-1 in the ischemic mouse heart associated with increased recruitment of CD45+/CXCR4-EGFP+/CD11b+ cell subsets. Enhanced PH inhibition significantly upregulated reparative M2 like CXCR4-EGFP+ CD11b+/CD206+ cells compared to inflammatory M2-like CXCR4-EGFP+ CD11b+/CD86+ cells associated with reduced apoptotic cell death, increased neovascularization, reduced scar size, and an improved heart function after MI. In summary, our data suggest increased PH inhibition as a promising tool for a customized upregulation of SDF-1 and CXCR4 expression to attract CXCR4+/CD11b+ cells to the ischemic heart associated with increased cardiac repair.

**Key messages:**

- DMOG-induced prolyl-hydroxylase inhibition upregulates SDF-1 and CXCR4 in human endothelial cells.
- Systemic application of DMOG upregulates nuclear HIF-1α and SDF-1 in vivo.
- Enhanced prolyl-hydroxylase inhibition increases mainly CXCR4+/CD11b+ cells.
- DMOG increased reparative M2-like CD11b+/CD206+ cells compared to M1-like cells after MI.
- Enhanced prolyl-hydroxylase inhibition improved cardiac repair and heart function.

**Electronic supplementary material:**

The online version of this article (doi:10.1007/s00109-017-1543-3) contains supplementary material, which is available to authorized users.

## Introduction

Ischemic cardiomyopathy due to acute myocardial infarction (MI) or coronary artery disease is a major contributor for mortality and morbidity in the Western world with rising incidence [1, 2]. Although pharmacological therapies can limit progression to chronic end-stage heart failure, repair of functional myocardium is very limited. Stromal cell-derived factor-1 (SDF-1) and its corresponding receptor CXCR4 have been shown to play prominent roles during cardiovascular development, cardiac repair, and tissue homeostasis after ischemia [3]. We could demonstrate that a dual therapy consisting of (stem) cell mobilization with granulocyte-colony stimulating factor (G-CSF) and stabilization of SDF-1 by preventing its cleavage through inhibition of the protease CD26 increased recruitment of blood-derived progenitor cells associated with attenuated post-MI remodeling, improved myocardial function, and increased survival in mice [4]. Combined G-CSF treatment with dipeptidyl peptidase-IV (DPP-IV) inhibition limiting the proteolytic degradation of SDF-1 plus cell cycle activation in cardiomyocytes even enhanced cardiac regeneration after MI [5]. SDF-1 is upregulated for only ca. 3 days after MI, limiting the targeting of circulating CXCR4+ progenitors to ischemic myocardium over a longer period of post-infarct remodeling [6]. Consequently, various groups have developed strategies to overexpress SDF-1 mRNA or deliver SDF-1 protein after acute myocardial infarction in the heart to attenuate ischemic cardiomyopathy [7–9]. As a hindrance, these strategies rely on invasive procedures with the risk for infections and bleeding. From a translational aspect, it would be desirable to customize SDF-1 and CXCR4 gene expression pharmacologically in a time-dependent manner. Hypoxia-inducible factor (HIF) prolyl-hydroxylases seem to be attractive tools to achieve this goal: SDF-1 and CXCR4 gene expression is regulated by HIF-1α, which can bind to both promoters [10, 11]. Since HIF-1α degradation is triggered by oxygen-sensitive hydroxylation of two conserved proline residues by HIF-prolyl-hydroxylases, inhibition of the latter may enforce SDF-1 and CXCR4 gene expression and stimulate cardiac repair in the ischemic heart. In line with that assumption, a recently published study suggests that early activation of CXCR4 in cardiomyocytes after a single intramyocardial injection of the HIF-prolyl-hydroxylase inhibitor DMOG improved myocardial function by preventing cardiomyocyte cell death [12]. However, this study neither analyzed HIF-1α activation and SDF-1 expression nor investigated recruitment of CXCR4+ cells from the bone marrow (BM) and heart. Therefore, we hypothesized that DMOG-induced PH inhibition may activate SDF-1 expression to augment CXCR4+ cell recruitment for cardiac repair.

## Methods (an expanded methods section is available in the online supplement)

### Surgical induction of myocardial infarction in mice

Myocardial infarction was induced in 10–12-week-old CXCR4-EGFP and C57BL/6 mice by surgical occlusion of the left anterior descending artery (LAD) through a left anterolateral approach as described previously [4]. Animal care and all experimental procedures are performed in strict accordance to the Austrian and National Institutes of Health animal legislation guidelines.

### Fluorescent-activated cell sorting (FACS) of BM and heart cells

Ten- to 12-week-old CXCR4-EGFP BAC transgenic reporter mice with or without LAD ligation were either treated with saline or DMOG (80 mg/kg/day) for up to 7 days. BM mononuclear and myocyte-depleted cardiac cells were separated as previously described [4]. Cells were incubated for 40 min in the dark at 4 °C with the following fluoresceinisothiocyanate (FITC)-, phycoerythrin (PE)-, and peridininchlorophyll-protein (PerCP)-conjugated monoclonal antibodies: CD45-PerCP, CD11b-PerCP, CD11b-PE, CD4-PE, CD20-PE, CD31-PE, CD34-PE, Flk-PE, CD86-PE, CD206-PE, F4/80-PE, CD133-PE, c-kit-PE, Sca-1-PE, CD3-biotin, CD45R/B220-biotin, CD11b-biotin, TER-119-biotin, and Ly-6G-biotin (all from BD Pharmingen). Matching isotype antibodies (BD Pharmingen) served as controls. Cells were analyzed by three-color flow cytometer using a Coulter Epics XL-MCLTM flow cytometer (Beckman Coulter). Each analysis included 50,000 events.

### Expression analyses of SDF-1 and CXCR4 mRNA in human HMEC-1, HAVSMC, and murine BM cells

Human microvascular endothelial cells (HMEC-1; CDC, Atlanta, GA) and human aortic vascular smooth muscle cells T/G HAVSMC (HAVSMC; American Type Culture Collection, Manassas, VA) were grown according to the manufacturer. For expression analyses in BM cells, DMOG-treated and control (C57BL/6J) mice were sacrificed and mononuclear BM cells were collected by Ficoll density gradient centrifugation. Total RNA was reverse-transcribed using the QuantiTect RT kit (Qiagen) according to manufacturer’s protocol. Exon spanning primers for human and mouse SDF-1, CXCR4, GAPDH, and β-actin were designed (Supplementary Table 1). Using 2× SYBR green mastermix (Applied Biosystems, USA), quantitative gene expression was calculated using the comparative ΔΔCt− method (for detailed protocol, see online supplement).

### Western blot

For HIF-1α studies cytosolic and nuclear fractions were isolated as described previously [13]. SDS-PAGE and blotting analysis were done using the following antibodies: HIF-1α (rabbit polyclonal against HIF-1α, ab82832 Abcam), TATA binding protein (TBP) (mouse monoclonal against TBP, ab51841, Abcam), and GAPDH (mouse monoclonal to GAPDH, ab8245, Abcam). Appropriate horseradish-peroxidase-coupled secondary antibodies were used, and chemiluminescence was performed with ECL-Prime (Amersham). Western blot replicates were scanned and quantified using ImageJ computer-assisted densitometric analysis. HIF-1α protein expression was normalized to GAPDH (cytosol) and TBP (nuclear compartment).

### Gene expression of HIF-1 target genes in the heart

Heart tissues were minced and homogenized in TRIzol reagent (Invitrogen, USA), and total RNA was isolated according to the manufacturer’s instructions. RNA was reverse-transcribed to cDNA using the QuantiTect RT kit (Qiagen). Exon spanning primers for the mouse HIF-1α target genes vascular endothelial growth factor A (VEGF-A), pyruvate dehydrogenase kinase 1 (PDK1), and lactate dehydrogenase A (LDHA) were designed and are listed in Supplementary Table 1. Using 2× SYBR green mastermix (Applied Biosystems, USA), quantitative gene expressions were calculated using the comparative ΔΔCt− method with β-actin as a reference gene.

### SDF-1 ELISA

A commercially available ELISA kit (R&D systems, Mouse CXCL12/SDF-1 alpha Quantikine ELISA Kit) was used to measure SDF-1 protein levels in heart lysates 7 days after myocardial infarction following manufacturer’s instructions.

### Histology, immunostaining, and quantification of apoptosis and neovascularization

Thirty days after MI, hearts (*n* = 10) were excised and fixed in 4% phosphate-buffered formalin and processed for analyses of scar size, wall thickness, capillary density, and TUNEL assay for apoptotic cells according to standard procedures (for detailed protocol, see online supplement) [14, 15]. Cardiomyocyte size was determined measuring myocyte cross-sectional area and minimum Feret’s diameter from Masson’s trichrome stained transverse sections at the periinfarct zone. Fifty cardiomyocytes per field were analyzed using ImageJ analysis software.

### Functional parameters

To evaluate functional parameters, mice were randomly assigned to the following groups: sham-operated control mice (*n* = 5), saline-treated infarcted mice (*n* = 10), and DMOG (80 mg/kg i.p.)-treated infarcted mice (*n* = 10). Pharmacological treatment was administered for up to 7 days post-MI (Supp. Fig. 1). Pressure-volume relations in vivo were analyzed 30 days after MI as previously described [4].

### Statistical analysis

Results were expressed as mean ± SD. Multiple-group comparisons were performed by one-way analysis of variance (ANOVA) followed by the Bonferroni procedure for comparison of means. Comparisons between two groups were performed using the unpaired two-sided Student’s *t*-test. Data were considered statistically significant at a value of *p* <0.05.

## Results

### Myocardial ischemia highly upregulates CD45+/CXCR4-EGFP+/CD11b+ cells in the BM and the heart

**Fig. 1:**
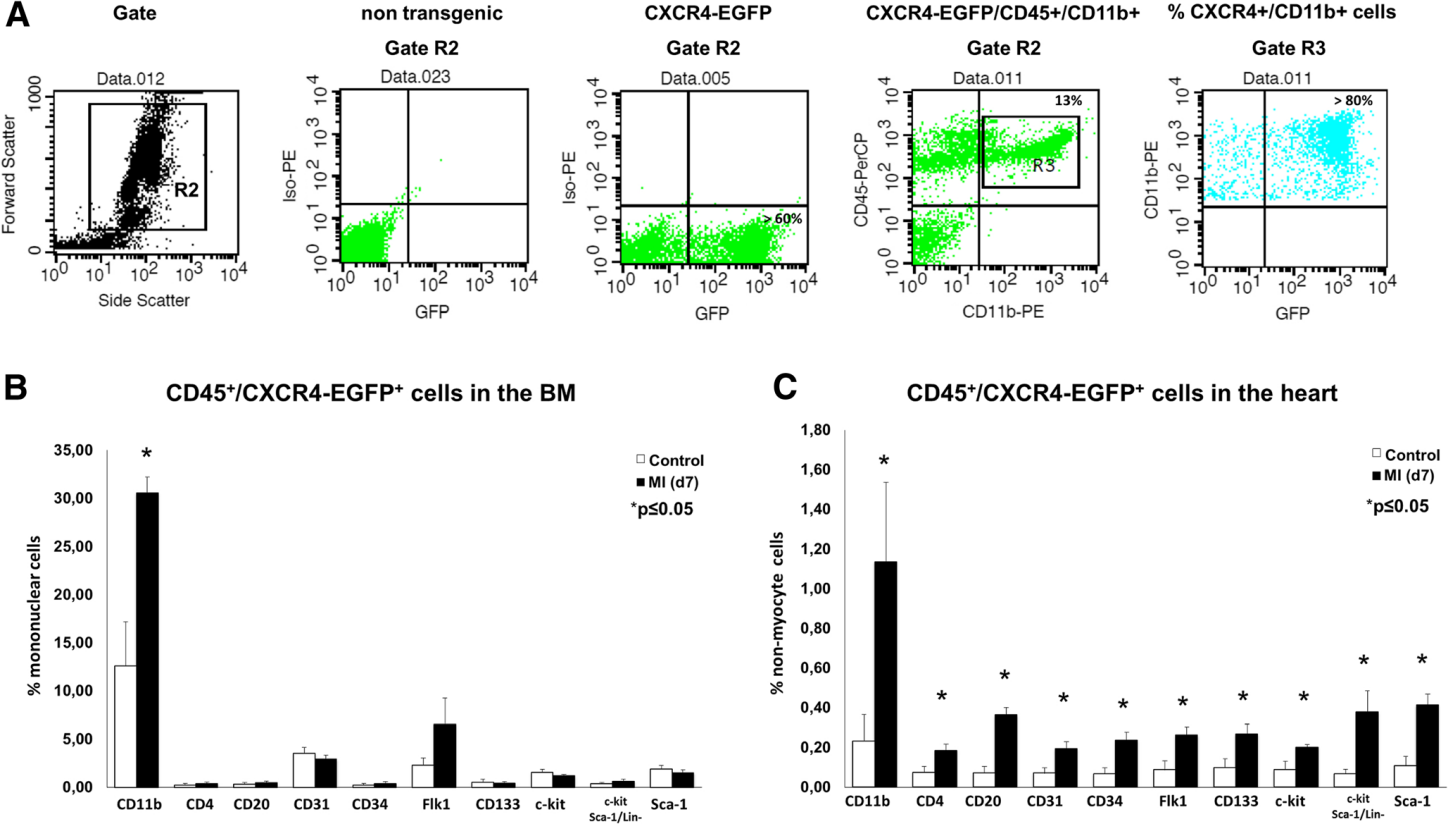
FACS analysis of CXCR4-EGFP reporter mice display enhanced numbers of CD45+/CXCR4+/CD11b+ cells in the BM and ischemic heart. **a** Representative FACS analysis of mononuclear BM cells from CXCR4-EGFP and non-transgenic littermate controls showing high expression of CXCR4-EGFP+ on CD45+/CD11b+ cells. **b**, **c** Bar graph showing numbers of CD45+/CXCR4-EGFP+ cells in the BM and heart at day 7 ± MI. All data represent mean ± SD (*n* = 4); *p < 0.05 control vs. MI

Transgenic CXCR4-EGFP reporter mice were utilized to analyze CD45+/CXCR4-EGFP+ and CD45−/CXCR4-EGFP+ cell populations from BM and heart under normoxia and ischemia. These mice contain multiple copies of a modified BAC in which the enhanced green fluorescent protein (EGFP) reporter gene was inserted immediately upstream of the coding sequence of CXCR4 [16]. Figure 1a shows representative FACS analyses of mononuclear BM cells from non-transgenic controls and CXCR4-EGFP reporter mice gated for leukocytes (gate R2). Roughly >60% of the gated BM cells stained positive for CXCR4-EGFP. Gating of CD45-PerCP and CD11b-PE-positive cells revealed that >80% of CD11b+ cells co-expressed CXCR4-EGFP. For further analyses, a broad panel of different leukocyte cell markers was used to screen for subsets of CXCR4-EGFP+ expressing cells. As shown in Fig. 1b, CD45+/CXCR4-EGFP+ was most frequently expressed in monocytic CD11b+ BM cells. Seven days after ischemia, particularly CD45+/CXCR4-EGFP+/CD11b+ cells significantly increased, whereas CD45−/CXCR4-EGFP+ cells were only rarely detected in BM and did not significantly change after MI (Supp. Fig. 2A). In the normoxic heart, CD45+/CXCR4-EGFP+ as well as CD45−/CXCR4-EGFP+ cells were very rarely detected (Fig. 1c and Supp. Fig. 2B). Seven days after MI, CD45+/CXCR4-EGFP+ cells co-expressing monocytic CD11b+, T-lymphocytic CD4+, B-lymphocytic CD20+, angiogenic CD31+, CD34+, c-kit+, and Flk1+ cells, as well as stem cell populations like CD133+, c-kit+, and Lin−/c-kit+/Sca-1+, and Sca-1+ were significantly increased (Fig. 1c), whereas CD45−/CXCR4-EGFP+ cells did not significantly change after MI (Supp. Fig. 2B). Further immunostainings confirmed that CXCR4-EGFP+ cells were almost absent under normoxic conditions in the heart (Supp. Fig. 3, first row). Seven days after ischemia, CXCR4-EGFP+ could be co-stained in PECAM (CD31)-positive endothelial cells and infiltrating CD11b+ monocytic cells (Supp. Fig. 3).

### DMOG upregulates SDF-1 and CXCR4 mRNA expression in human endothelial and smooth muscle cells in vitro and murine BM in vivo

**Fig. 2:**
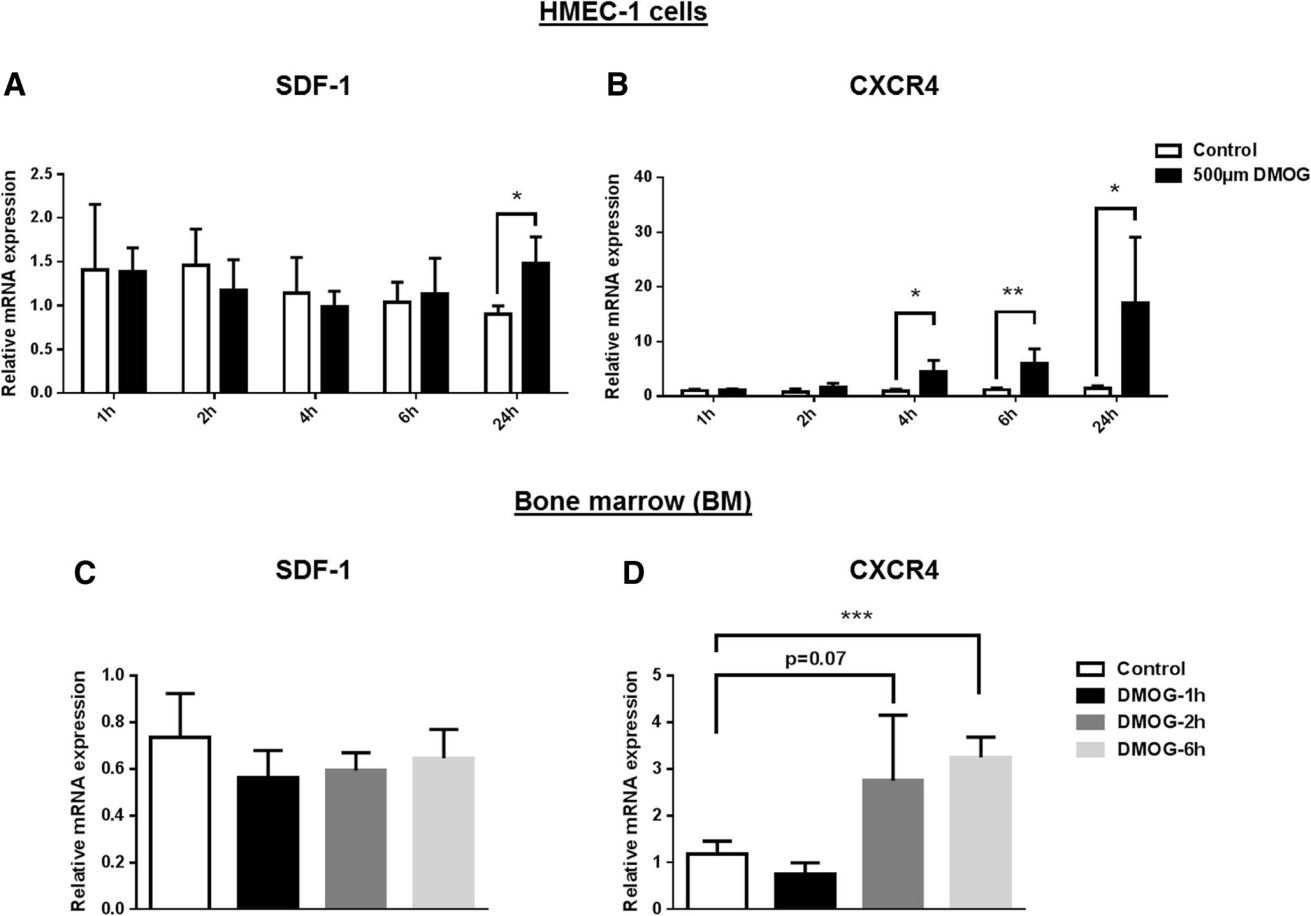
DMOG increases SDF-1 and CXCR4 mRNA expression in a time- and dose-dependent manner in vitro and in vivo. **a**, **b** Expression of SDF-1 and CXCR4 mRNA in human endothelial HMEC-1 cells in vitro treated with 500 μM of DMOG for 1, 2, 4, 6, and 24 h. Data represent mean ± SD (*n* = 6); *p < 0.05, **p < 0.01 control vs. DMOG. **c**, **d** Expression of SDF-1 and CXCR4 mRNA in murine mononuclear BM cells in vivo treated with 80 mg/kg of DMOG i.p. for 1, 2, and 6 h. Data represent mean ± SD (*n* = 4); ***p < 0.001 control vs. DMOG

Since SDF-1 and CXCR4 are known HIF-1α target genes [10, 11], we hypothesized that DMOG-induced PH inhibition upregulates SDF-1 and CXCR4 mRNA expression in endothelial, smooth muscle, and BM cells known to express either SDF-1 or CXCR4 [3, 17]. In vitro, cultivated human endothelial HMEC-1 and aortic vascular smooth muscle HAVSMC cells were treated with 500 μM of DMOG known to induce HIF-1 target gene expression [18]. SDF-1 as well as CXCR4 mRNA expression was analyzed 1, 2, 4, 6, and 24 h after DMOG administration. SDF-1 mRNA expression was significantly elevated in human endothelial HMEC-1 cells 24 h after treatment (Fig. 2a). CXCR4 was upregulated earlier starting 4 h after DMOG treatment reaching a maximum after 24 h (Fig. 2b). Other known HIF-1α target genes like VEGF-A, PDK1, and LDHA were also significantly upregulated after DMOG treatment in HMEC-1 cells (Supp. Fig. 4). In HAVSMC smooth muscle cells, SDF-1 mRNA was upregulated 24 h after DMOG treatment (Supp. Fig. 5A), whereas CXCR4 was almost undetectable (Supp. Fig. 5B).

In vivo, mice were treated with 80 mg/kg DMOG i.p. for 1, 2, and 6 h and analyzed for SDF-1 and CXCR4 mRNA expression in mononuclear BM cells. In contrast to SDF-1 mRNA, CXCR4 was significantly upregulated after 6 h of DMOG treatment (Fig. 2c, d).

### Upregulation of nuclear HIF-1α and SDF-1 in the heart after PH inhibition in vivo

**Fig. 3:**
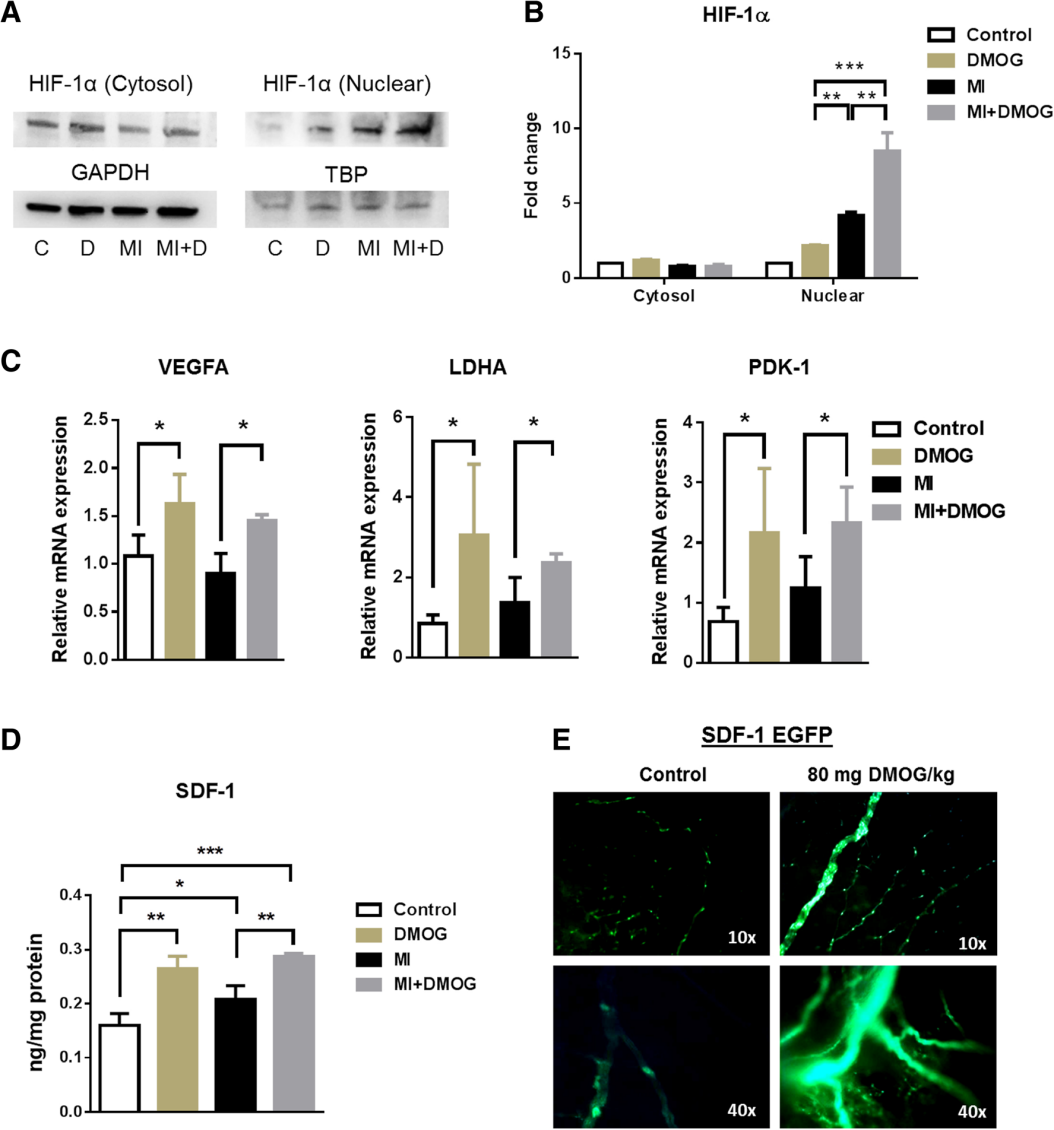
Prolonged HIF-1α and SDF-1 upregulation after DMOG treatment. **a** HIF-1α expression in the cytosol and the nuclear fraction of non-infarcted (C), non-infarcted + DMOG (D), infarcted (MI), and infarcted + DMOG (MI + D)-treated mouse hearts determined by Western blot. DMOG was administered for 7 days. **b** Quantitative densitometric analysis of Western Blot replicates normalized to GAPDH (cytosol) and TBP (nuclear compartment). **c** Expression of HIF-1α target genes VEGF-A, PDK1, and LDHA in non-infarcted and infarcted hearts ± DMOG for 7 days. **d** ELISA for SDF-1 protein in heart lysates of non-infarcted and infarcted animals showed a significant upregulation of SDF-1protein in hearts of DMOG treated mice after 7 days of treatment. **e** SDF1-EGFP reporter mice treated with the prolyl-hydroxylase inhibitor DMOG (80 mg/kg, i.p.) showed a robust induction of SDF1-EGFP activity in coronary artery vessels and capillary networks in the heart. All data represent mean ± SD (*n* = 3); *p < 0.05; **p < 0.01; ***p < 0.001

Next, we determined whether DMOG is capable of inducing HIF-1α and SDF-1 expression in the heart in vivo. HIF-1α expression was determined in the cytosol and the nuclear fraction in non-infarcted and infarcted mouse hearts ± DMOG administration by Western blot 7 days after MI. As shown in Fig. 3a, b, DMOG treatment significantly increased HIF-1α expression in the nuclear but not in the cytosolic compartment in non-infarcted (D) and infarcted animals (MI + D), suggesting that HIF-1α has translocated to the nucleus to form its transcriptionally active compound. Other known HIF-1α target genes like VEGF-A, PDK1, and LDHA were also significantly upregulated after 7 days of DMOG treatment in the mouse heart (Fig. 3c). Since SDF-1 is known to be downregulated from day 3 to day 7 after MI, we examined whether DMOG-induced PH inhibition leads to an upregulation of SDF-1 in the ischemic heart at later time points. As shown in Fig. 3d, DMOG treatment induced a significant upregulation of SDF-1 not only in non-infarcted but also in infarcted mouse hearts 7 days after MI. Furthermore, non-infarcted SDF-1-EGFP reporter mice were treated with 80 mg/kg of DMOG i.p. for 48 h (*n* = 3 animals per group). Thereafter, hearts were excised and investigated under a fluorescence microscope for SDF-1-EGFP reporter activity. Figure 3e reveals a pronounced activation of SDF1-EGFP in non-infarcted hearts after DMOG-induced PH inhibition compared to saline-treated controls.

### DMOG predominantly increased CD45+/CXCR4-EGFP+/CD11b+ cells after MI

**Fig. 4:**
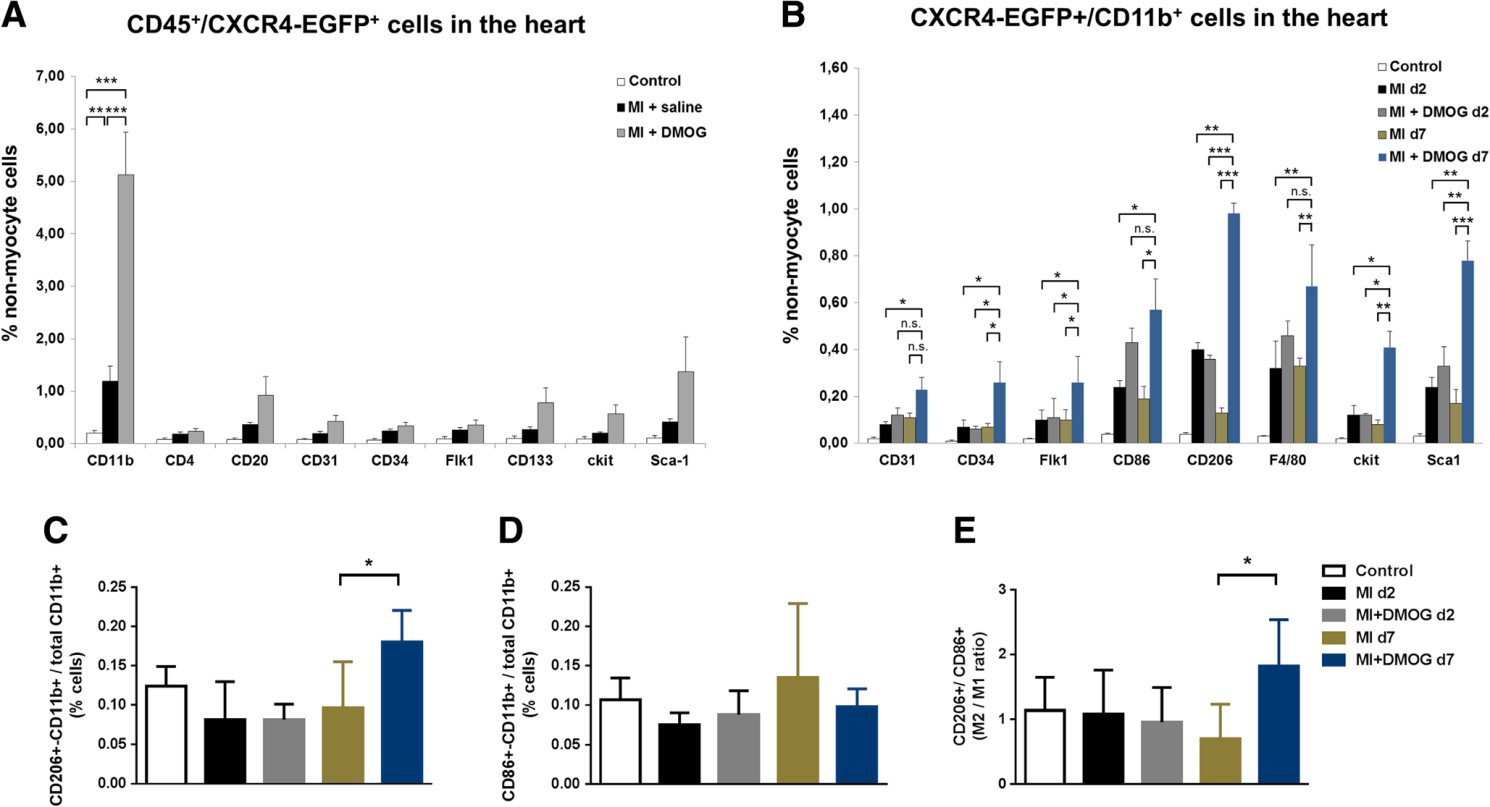
DMOG increased CD45+/CXCR4-EGFP+ cells in the ischemic heart. **a** FACS analyses of CXCR4-EGFP reporter mice showed increased numbers of CD45+/CXCR4-EGFP+/CD11b+ cells in the ischemic heart after DMOG treatment. **b** Further analyses of CXCR4+/CD11b+ subpopulations revealed an increased expression of Sca1+, CD86+, CD206+, and F4/80+ cells particularly 7 days after PHI. **c** Bar graph representing the number of CXCR4-EGFP+ CD206+/CD11b+ to total CXCR4-EGFP+ CD11b+ cells. **d** Bar graph showing the number of CXCR4-EGFP+ CD86+/CD11b+ to total CXCR4-EGFP+ CD11b+ cells. **e** Bar graph showing the ratio of CXCR4-EGFP+ CD11b+/CD206+ cells to CXCR4+-EGFP+ CD11b+/CD86+ cells in non-infarcted control and infarcted hearts ± DMOG treatment for 2 and 7 days, respectively. Data represent mean ± SD (*n* = 6); *p < 0.05; **p < 0.01; ***p < 0.001

Since DMOG-induced PH inhibition prolonged SDF-1 upregulation in the infarcted heart for 7 days, we analyzed CXCR4-EGFP+ cells in the ischemic heart. Therefore, non-infarcted and infarcted CXCR4-EGFP reporter mice were treated with saline or DMOG, respectively (Supp. Fig. 1). Seven days after MI, hearts were excised and analyzed for CXCR4-EGFP+ and co-expression of the leukocyte common antigen CD45 by FACS. As shown in Fig. 4a, DMOG treatment revealed a significant upregulation of CD45+/CXCR4-EGFP+/CD11b+ monocytic cells after MI. CD45+/CXCR4-EGFP+ cells co-expressing T-lymphocytic CD4, B-lymphocytic CD20, angiogenic CD31, CD34, Flk-1, as well as stem cell markers like CD133, c-kit, and Sca-1 were not significantly increased after enhanced PH inhibition with DMOG. CD45−/CXCR4-EGFP+ cells were very rarely detected and did not significantly change after MI ± DMOG (Supp. Fig. 6).

### DMOG highly increased the ratio of CD11b+/CD206+ compared to CD11b+/CD86+ cells in the ischemic heart

Since CD11b+ cells are known to enhance angiogenesis [19] and play a major role in inflammatory and reparative processes after tissue injury, further potentially angiogenic subsets of CD11b+ cells were analyzed at day 2 and day 7 after MI ± DMOG treatment, reflecting the early and late phase of inflammatory response after myocardial infarction with the major impact on cell infiltration [20, 21]. Endothelial markers like CD31, CD34, Flk-1, macrophages markers for M1 type inflammatory CD86, M2-like reparative CD206 and F4/80 cells, as well as stem cell markers like c-kit and Sca-1 were used to further characterize CD11b+ cell subsets in vivo. As shown in Fig. 4b, increased PH inhibition with DMOG significantly upregulated CXCR4+/CD11b+ cells co-expressing angiogenic CD34, Flk-1, M1 like CD86, M2 like CD206, F4/80, and stem cell markers like c-kit and Sca-1 particularly at 7 days but not at 2 days after MI. Next, we calculated the ratio of reparative M2-like CXCR4-EGFP+ CD206+CD11b+ cells to total CXCR4-EGFP+ CD11b+ cells (Fig. 4c) as well as the ratio of inflammatory M1-like CXCR4-EGFP+ CD86+CD11b+ to total CXCR4-EGFP+ CD11b+ cells (Fig. 4d). As shown in Fig. 4c, DMOG treatment significantly increased the subset of CD206+ cells within the CD11b+ cell population of CXCR4-EGFP+ cells. Moreover, DMOG treatment for 7 days after MI significantly increased the ratio of CXCR4-EGFP+ CD11b+/CD206+ cells to CD11b+/CD86+ cells by 2.6-fold from 0.7 to 1.82 compared with infarcted control animals, suggesting an improved fine-tuning of reparative M2-like CD206+ compared to inflammatory CD86+ cells in the ischemic heart (Fig. 4e).

### Prolyl-hydroxylase inhibition reduced apoptotic cells and increased neovascularization

**Fig. 5:**
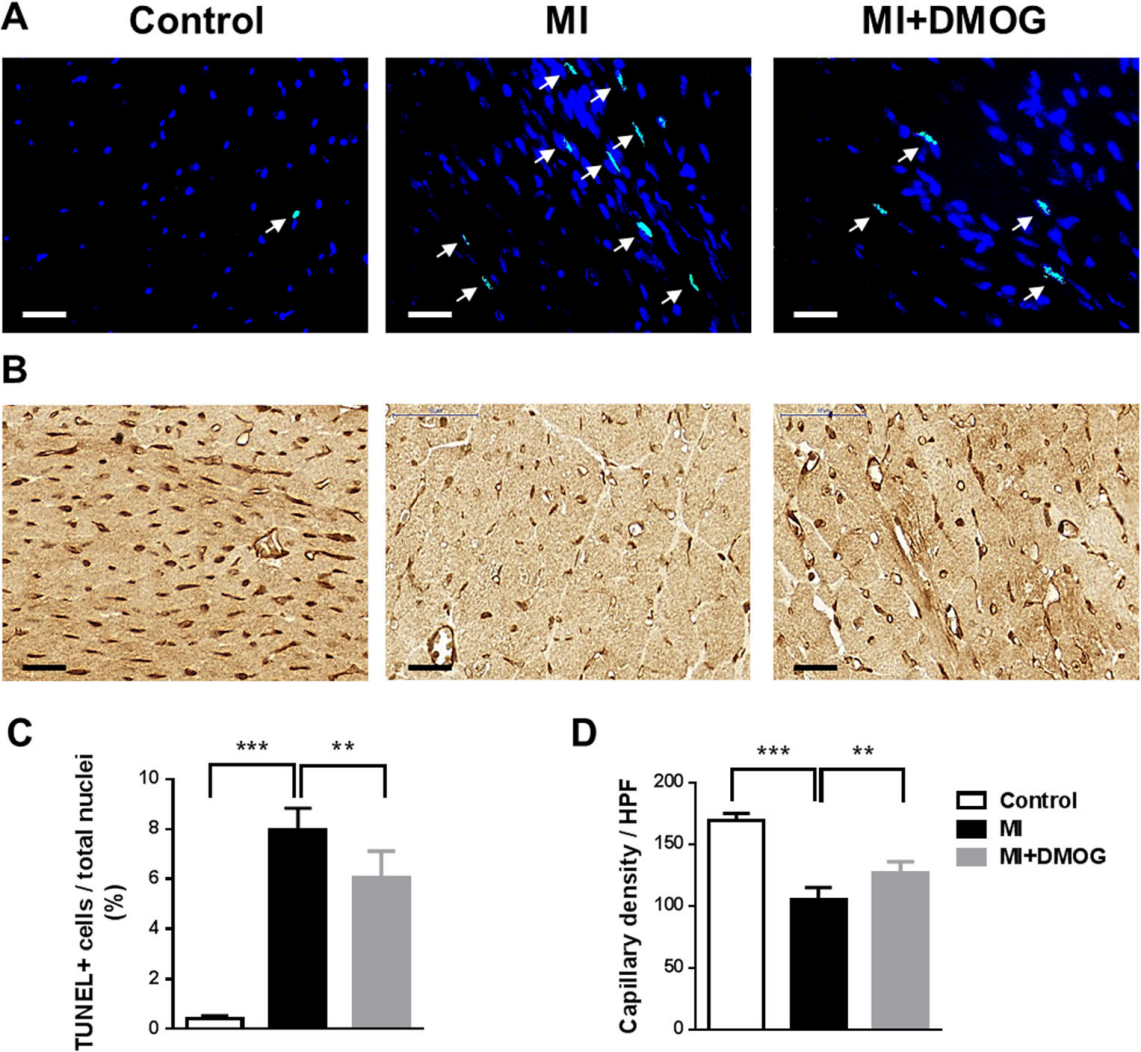
DMOG treatment decreased apoptotic cells and increased neovascularization. **a** Representative co-staining of TUNEL-positive (*bright green nuclei marked by arrows*) and DAPI+ (*blue nuclei*) cells in non-infarcted and infarcted mice ± DMOG treatment 30 days after MI. *Scale bar* represents 25 μM. **b** Representative immunohistochemical staining of CD31 (*brown*) in infarcted hearts 30 days after MI. *Scale bar* represents 25 μM. **c** Bar graph representing the percentage of TUNEL-positive nuclei to total nuclei in the infarct border zone 30 days after MI. Data represent mean ± SD (*n* = 6). **d** Bar graph showing the numbers of CD31+ capillaries in the infarct border zone in non-infarcted control mice, infarcted mice, and infarcted mice treated with DMOG 30 days after MI. Data represent mean ± SD (*n* = 6); **p < 0.01, ***p < 0.001 (color figure online)

Since PH-inhibitor treatment was administered for 7 days, we analyzed apoptotic cell death and neovascularization at a later time point 4 weeks after MI. Hearts were excised and histologically examined for apoptotic cells by TUNEL assay. Apoptotic index (AI) was calculated as percentage of apoptotic nuclei/total nuclei. AI in DMOG-treated mice was significantly reduced compared to saline-treated infarcted animals (6.03 ± 1.08 vs. 8.00 ± 0.86, *p* ≤ 0.001; Fig. 5a, c). Neovascularization was quantified by counting CD31+ vessels in the periinfarct region. As shown in Fig. 5b, d, numbers of CD31+ vessels were significantly increased after treatment with DMOG compared to saline-treated infarcted animals (127.1 ± 3.6 vs. 105 ± 4.1, *p* ≤ 0.002).

### DMOG treatment reduced scar size and improved myocardial function

**Fig. 6:**
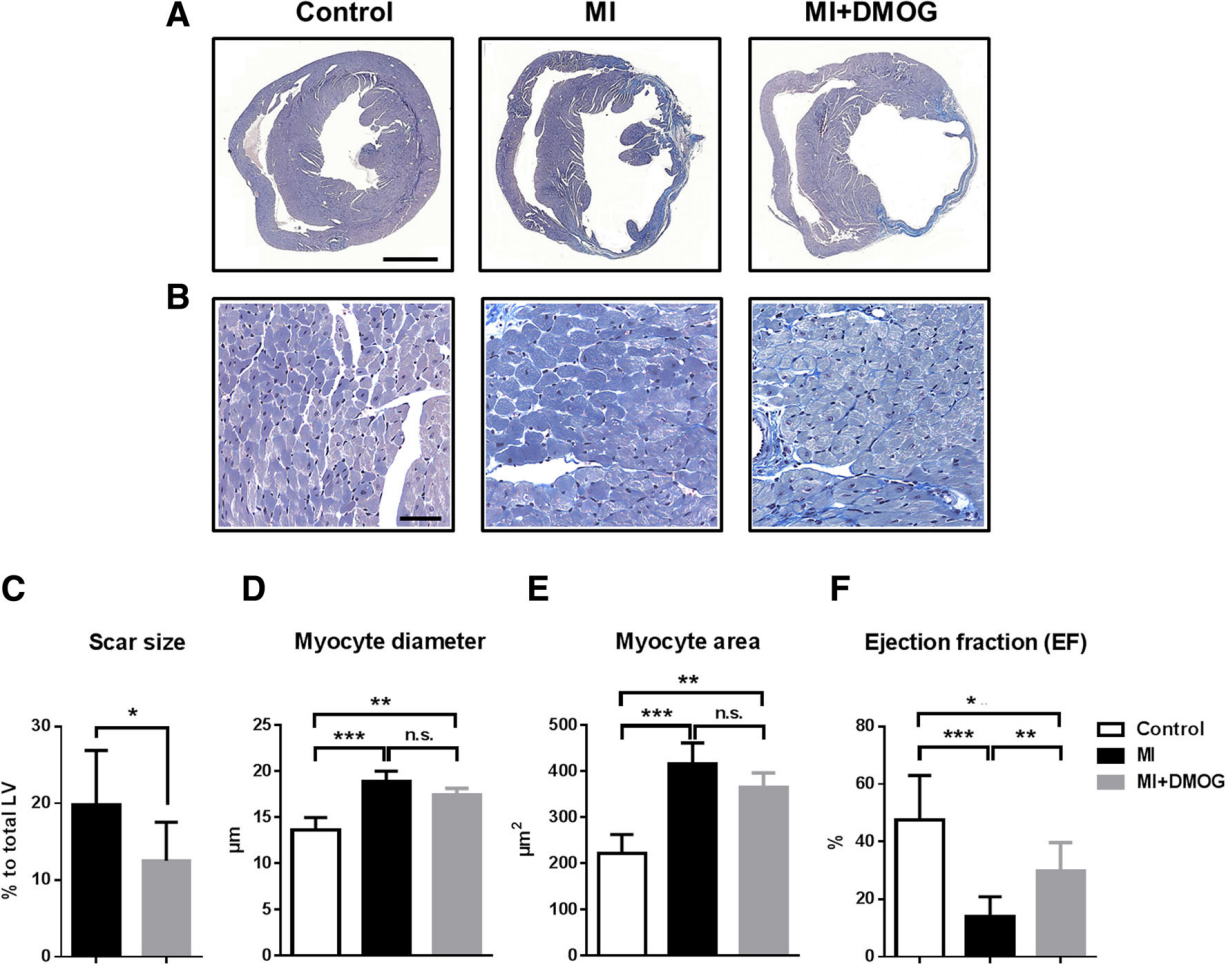
PH inhibition with DMOG reduced scar size and improved cardiac function. **a** Representative Masson’s trichrome stainings of heart sections analyzed 4 weeks after MI. *Scale bar* represents 1 mm. **b** Massons’s trichrome stainings from peri-infarct region 4 weeks after MI of control, infarcted (MI), and infarcted + DMOG-treated (MI + DMOG) animals. *Scale bar* represents 50 μM. **c** Infarct size was significantly reduced in the DMOG-treated group compared to saline-treated infarcted mice. **d** Bar graphs representing the minimum Feret’s diameter and **e** cross-sectional area of cardiomyocytes in the infarct border zone of control, infarcted (MI), and infarcted + DMOG-treated (MI + DMOG) animals. **f** Diagram representing ejection fraction in sham (*n* = 5), saline + MI (*n* = 10), and DMOG + MI (*n* = 10) treated groups. All data represent mean ± SD; *p < 0.05, **p < 0.01, ***p < 0.001. *n.s*. not significant

Since DMOG treatment reduced apoptotic cell death and increased neovascularization, we analyzed important determinants for cardiac remodeling like scar size, hypertrophic response, and heart function. Four weeks after MI, hearts were excised and histologically examined for infarct scar size and wall thickness. Scar size in DMOG-treated mice was significantly smaller compared to saline-treated infarcted animals (12.5 ± 2.5 vs. 19.8 ± 2.4%, *p* < 0.05; Fig. 6a, b) although expression of the fibrosis marker collagen-1a was not significantly different 7 days after MI (Supp. Fig. 7A). The thickness of the infarcted and non-infarcted left ventricular wall was not significantly different between the two groups (infarct site 0.34 ± 0.16 vs. 0.32 ± 0.07 mm, *p* = 0.92; periinfarct site 1.13 ± 0.18 vs. 1.10 ± 0.08 mm, *p* = 0.87). Compared to control, infarcted mice ± DMOG treatment revealed a significantly increased minimum Feret’s diameter and cross-sectional area of cardiomyocytes in the periinfarct region as signs of hypertrophic response after MI. Although there was a tendency to decreased values after DMOG administration, we found no significantly difference between infarcted animals ± DMOG treatment (Fig. 6b, d, e). Furthermore, there was no difference in the expression of the hypertrophy marker brain natriuretic peptide (BNP) between DMOG and non-treated infarcted animals 7 days after MI (Supp. Fig. 7B).

To analyze hemodynamic effects of DMOG treatment, pressure volume relations were measured in vivo from sham-operated, saline-treated infarcted, or DMOG-treated infarcted mice, respectively (Table 1 and Fig. 6f). Compared to saline MI, DMOG treatment revealed a significantly improved systolic function, reflected by an increased ejection fraction (29.69 ± 9.95 vs. 14.12 ± 6.81%, *p* < 0.01; Fig. 6f). Other important hemodynamic parameters like stroke volume, cardiac output, and stroke work were also significantly improved in DMOG-treated mice (Table 1). Furthermore, increased PH inhibition with DMOG revealed attenuated ventricular dilation, measured by end-diastolic volumes (Table 1).

**Table 1:** Mouse hemodynamic measurements

Hemodynamic parameters	Control (*n* = 5)	MI (*n* = 10)	MI + DMOG (*n* = 10)
HR (bpm)	343 ± 82	326 ± 89	347 ± 91
Ves (μL)	16.46 ± 7.12	38.97 ± 9.68	22.73 ± 10.91**
Ved (μL)	30.98 ± 7.66	44.82 ± 8.55	31.47 ± 12.44*
Pes (mmHg)	92.98 ± 18.61	86.26 ± 11.15	87.05 ± 21.01
Ped (mmHg)	8.88 ± 1.65	11.72 ± 7.00	6.48 ± 5.10
SV (μL)	14.52 ± 5.76	5.85 ± 1.64	8.74 ± 3.26*
EF (%)	47.46 ± 15.56	14.12 ± 6.81	29.69 ± 9.95**
CO (μL/min)	5166 ± 2547	1963 ± 953	3046 ± 1299*
SW (mmHg/μL)	1251 ± 314	451 ± 104	806 ± 335*
Ea (mmHg/μL)	6.39 ± 2.83	12.39 ± 3.74	9.34 ± 2.58
Tau (ms)	12.07 ± 3.71	12.38 ± 3.41	9.22 ± 2.52

Values are mean ± SD

*HR* heart rate, *Ves* end-systolic volume, *Ved* end-diastolic volume, *Pes* end-systolic pressure, *Ped* end-diastolic pressure, *SV* stroke volume, *EF* ejection fraction, *CO* cardiac output, *SW* stroke work, *Ea* arterial elastance, *Tau* time constant for isovolumic relaxation.

*p < 0.05 MI vs. MI + DMOG; **p < 0.01 MI vs. MI + DMOG

## Discussion

**Fig. 7:**
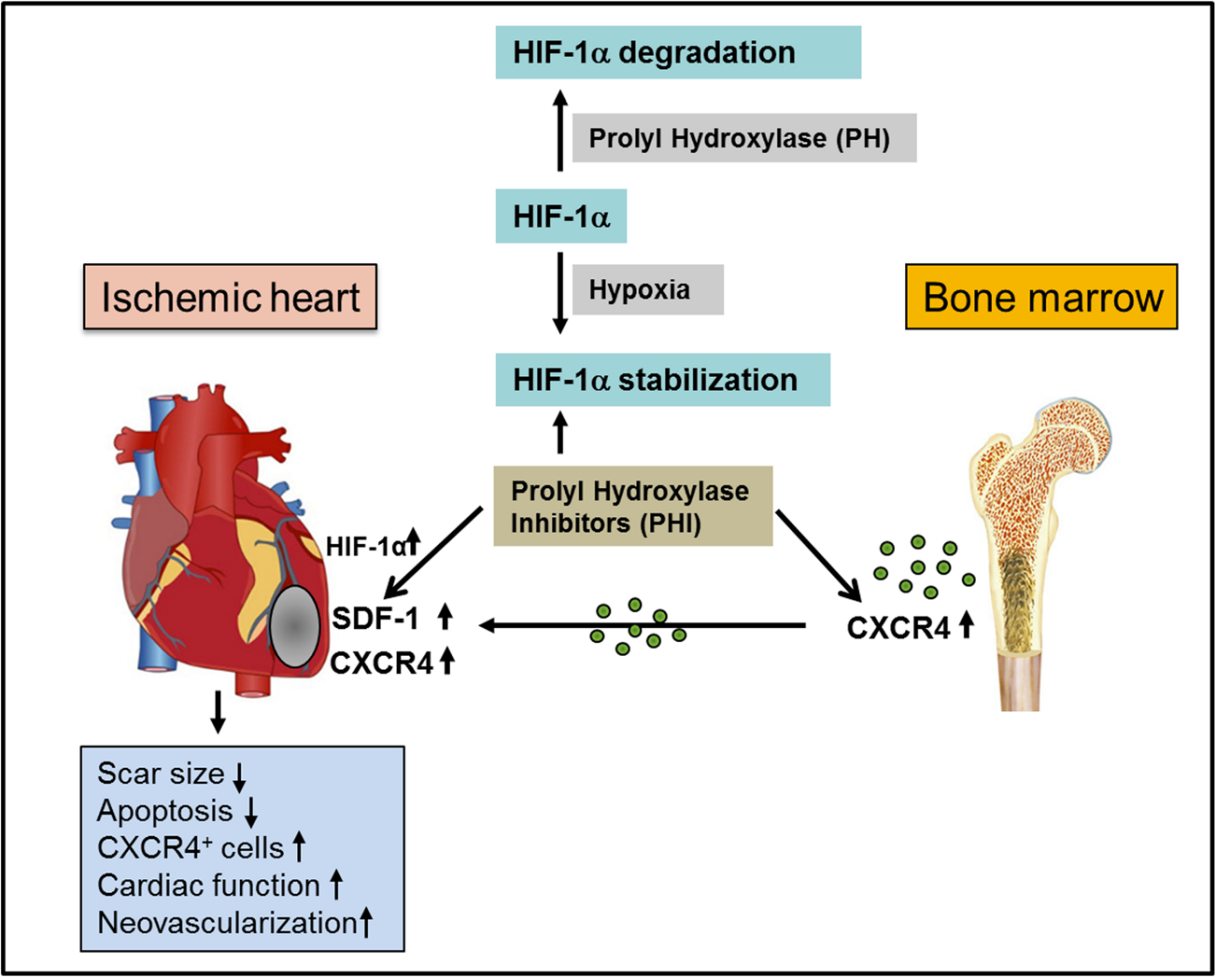
Therapeutic concept of prolyl-hydroxylase inhibition to enhance SDF-1 and CXCR4 expression for myocardial repair. Under normoxic conditions, HIF-1α protein is inactivated by prolyl-hydroxylases leading to HIF-1α degradation. Hypoxia or PHI leads to a reduced prolyl-hydroxylases activity, thereby stabilizing HIF-1α, which translocates to the nucleus and upregulates downstream target genes like SDF-1 and CXCR4. HIF-1α dependent SDF-1 upregulation lasts only for up to 3 days after MI. In order to prolong and upregulate the expression of the HIF-1a target genes SDF-1 and CXCR4, pharmacological induced inhibition of prolyl-hydroxylases can be exploited to increase CXCR4+ cells in myocardial repair associated with reduced apoptotic cell death, increased neovascularization, reduced infarct size, and improved myocardial function

We show for the first time that systemic application of the prolyl-hydroxylase inhibitor DMOG after MI (1) upregulates nuclear HIF-1α and SDF-1 in the ischemic mouse heart in vivo associated with increased (2) recruitment of mainly CD45+/CXCR4-EGFP+/CD11b+ cell subsets. DMOG treatment increased the ratio of CXCR4-EGFP+ CD11b+/CD206+ to CD11b+/CD86+ cells by 2.6-fold, suggesting an improved fine-tuning of reparative M2-like CD206+ compared to inflammatory CD86+ cells in the ischemic heart associated with (3) reduced apoptotic cell death, increased neovascularization, reduced scar size, and (4) an improved heart function after MI (Fig. 7). SDF-1 and CXCR4 gene expression during ischemia is regulated by HIF-1α [10, 11]. Since SDF-1 is only upregulated for up to 72 h after MI [6], various groups have implemented strategies to overexpress SDF-1 mRNA or deliver SDF-1 protein [6–9]. However, these studies are based on invasive open chest protocols, whereas our study provides the clinical perspective that a non-invasive systemic application of a prolyl-hydroxylase inhibitor could upregulate SDF-1 and other HIF-1α target genes to improve cardiac repair. Our results are in line with previous studies showing that preconditioning with DMOG can protect hearts from ischemic damage and improve cardiac function [22, 23]. A recently published study even showed that mice exposed to systemic hypoxemia for 2 weeks after MI revealed reactivation of cardiomyocyte mitosis [24]. Studies investigating the effect of prolyl-hydroxylase inhibition after the ischemic event also required open chest protocols with intracardiac injections of short hairpin RNAs or DMOG. One study showed that short hairpin RNA silencing of prolyl-hydroxylase enhances stem cell migration associated with improved angiogenesis and heart function after MI [25, 26]. However, effects on SDF-1/CXCR4-mediated cell recruitment were not investigated. Another recently published study suggests that early activation of CXCR4 in cardiomyocytes after intramyocardial injection of DMOG improved myocardial function by preventing cardiomyocyte cell death [12]. In line with these reports, we also found reduced numbers of apoptotic cells and increased neovascularization even at later time points after DMOG treatment. Of note, our study differs from Mayorga et al. in several important points: DMOG was injected after MI directly into the heart by a single injection, whereas we administered the drug systemically for up to 7 days after MI. Neither HIF-1α upregulation nor expression of SDF-1 or any other HIF-1 target gene except CXCR4 was analyzed. Although CXCR4 expression was upregulated in the heart, CXCR4+ cell populations in BM and heart after MI ± DMOG treatment were not investigated. They also did not show a reduction in scar size after DMOG treatment. Thus, our data extends the current knowledge on the impact of increased PH inhibition on HIF-1α triggered activation of the cellular SDF-1/CXCR4 axis. Up to date, it is not known which subpopulation of CXCR4+ cells predominantly orchestrates infarct healing. Our CXCR4-EGFP cell tracking model utilizing a broad panel of different leukocyte cell markers revealed that particularly the subpopulation of CD45+/CXCR4+/CD11b+ cells was highly upregulated by ischemia ± DMOG treatment, suggesting a prominent role in CXCR4/SDF-1-mediated repair mechanisms. CD11b non-covalently associates with integrin β2 (CD18) and is expressed on granulocytes, monocytes/macrophages, dendritic cells, NK cells, and subsets of T and B cells [27]. CD11b/CD18 is critical for the transendothelial migration of monocytes and neutrophils. It is also involved in granulocyte adhesion, phagocytosis, and neutrophil activation [27]. Indeed, CD11b has been linked to enhanced angiogenesis by the secretion of angiogenic growth factors and support of sprouting and proliferation of endogenous endothelial cells [19]. Moreover, a recently published study suggests that CD11b+ myeloid cells infiltrating the heart are capable of secreting leucine-rich a2-glycoprotein attenuating myocardial fibrosis and increasing angiogenesis [28]. Of note, the ratio of M2-like CXCR4-EGFP+CD11b+/CD206+ cells to inflammatory M1-like CD11b+/CD86+ cells increased by 2.6-fold after DMOG-induced PH inhibition compared to infarcted control mice, suggesting an improved fine-tuning of reparative M2-like cells in the ischemic heart after 7 days. In line with that observation, CD206+ cells have been reported to support myocardial repair whereas the subpopulation of CD86+ cells reflects the early phase of inflammation which is involved in clearing of cell debris [29]. Depletion of CD206+ M2 macrophages by a CSF-1R kinase inhibitor was associated with decreased ventricular function, infarct enlargement, and increased inflammatory cell infiltration [30]. Recently published data even showed that depletion of CD11b+/CD206+ M2-like macrophages in mice lacking the kinase TRIB1 resulted in a catastrophic prognosis with frequent cardiac ruptures, as the result of markedly reduced collagen fibril formation in the infarct area due to impaired fibroblast activation. M2 like CD11b+/CD206+ cells also expressed higher levels of anti-inflammatory and repair-associated genes like interleukin 10 and VEGFA compared to M1 like macrophages. Decreased tissue repair and impaired cardiac function could be rescued by intrapericardial injection of 2 × 105 BM derived M2-like macrophages [31]. In our study, the M2/M1 ratio increased by 2.6-fold and numbers of CXCR4+/CD11b+/CD206+ cells were upregulated >7-fold after DMOG treatment for 7 days after MI. Based on previous data, we estimate that the intact adult mouse heart contains around 7 × 106 non-myocytes [32, 33] which can rise ca. 10-fold due to cell infiltration and proliferation after MI [20, 34, 35]. A fraction of 1% CXCR4+/CD11b+/CD206+ cells in a total-heart non-myocyte suspension after MI and DMOG treatment would mean around 7 × 10^5^ (10 × 7 × 10^6^ × 0.01) CXCR4+/CD11b+/CD206+ cells exceeding the numbers of injected reparative M2-like macrophages (2 × 10^5^) by far. Thus, increasing numbers of CXCR4+/CD11b+/CD206+ cells after MI with DMOG treatment could have a major biologically impact in the heart. Since increased PH inhibition with DMOG induced SDF-1 upregulation for 7 days after MI, the shift in favor of CD206 M2 like cells might be explained by an enhanced migration capacity towards SDF-1 compared to M1 like macrophages as described previously [36]. Furthermore, SDF-1/CXCR4 interactions might promote M2 polarization [37, 38]. Since CD11b is also a surface marker for neutrophils the latter may have facilitated post-infarction healing by polarizing macrophages towards a reparative M2c phenotype [39]. Improvement of heart function in our study could be explained by several reasons: scar size and end-diastolic volumes were reduced reflecting attenuated infarct remodeling; second perfusion and contractile tissue might be preserved due to increased capillary density and reduced apoptotic cell death in the periinfarct region. This effect might be related to SDF-1-mediated activation of the survival factor protein kinase B (PKB/Akt) known to increase neovascularization and reduce cardiomyocyte apoptosis [40]. Third, we found an increased M2/M1 ratio associated with improved cardiac function and survival as mentioned previously [31]. Moreover, CXCR4-EGFP+/CD11b+ cells co-expressing angiogenic markers like CD34, Flk1, or stem cell markers like c-kit and Sca-1 might have also contributed to improved neovascularization [41].

### Limitations of the study

One of the major limitations of our study is that we cannot rule out the possibility that DMOG activation of other HIF-1 target genes such as VEGF or EPO might have contributed to the observed benefits [23]. Also, DMOG-related mechanisms which have been described in terms of preconditioning like induction of protective endoplasmic reticulum stress genes [42] or prevention of activation of the ATR/CHK1/p53 pathway to decrease apoptosis might also play a role in cardioprotection [43]. With respect to the study design, we could have conducted apoptosis assays at an earlier time point and could have complemented functional assessment by echo data. The detailed mechanism why the ratio of M2/M1 shifted after PH inhibition and the role of other CXCR4+/CD11b+ cell populations remains to be resolved in future studies. Nevertheless, our study assumes an additional mechanism of PH inhibitor induced upregulation of nuclear HIF-1α and SDF-1 to stimulate CXCR4+/CD11b+ cell recruitment for cardiac repair. Since DMOG may not have the proper pharmacological profile for use in humans [44], other prolyl-hydroxylase inhibitors like FG-4592, which has recently been shown to corrected anemia in hemodialysis patients, may be applicable to clinical practice [45].

Our data suggest increased PH inhibition as a promising tool for a customized upregulation of SDF-1 and CXCR4 expression to attract reparative CXCR4+/CD11b+ cells to the ischemic heart associated with increased cardiac repair.

## Electronic supplementary material

ESM 1 (PDF 849 kb).
