# Supplementary material for: Prolyl-hydroxylase inhibition induces SDF-1 associated with increased CXCR4+/CD11b+ subpopulations and cardiac repair
Source: J Mol Med (Berl). 2017 May 26;95(8):825–37. doi: 10.1007/s00109-017-1543-3 (PMC5516048; doi:10.1007/s00109-017-1543-3)
Supplement: Supplementary file 1 — (PDF 849 kb). [file 109_2017_1543_MOESM1_ESM.pdf]

Electronic supplementary material (ESM)

J Mol Med 2017

Title: Prolyl-hydroxylase inhibition induces SDF-1 associated with increased CXCR4+/CD11b+ subpopulations and cardiac repair

Santhosh Kumar Ghadge, PhD [1], Moritz Messner, MD [1], Thi Van Pham MD [2], Maximilian Doppelhammer MD [2], Andreas Petry [3], Agnes Goerlach MD [3], Britta Husse PhD [1], Wolfgang-Michael Franz MD [1], Marc-Michael Zaruba MD [1]

1. Medical University Innsbruck, Department of Internal Medicine III, Cardiology and Angiology
2. Ludwig Maximilians University, Klinikum Grosshadern, Medical Department I
3. TU Munich, Department of Pediatric Cardiology and Congenital Heart Disease, Experimental Pediatric Cardiology. German Heart Center Munich

Corresponding author:

Ass.-Prof. PD Dr. Marc-Michael Zaruba
Medical University Innsbruck
Department of Internal Medicine III
Cardiology and Angiology
Anichstr. 35
6020 Innsbruck
Email: Marc-Michael.Zaruba@i-med.ac.at
Telephone: +43 512 504 23046
Fax: +43 512 504 23169

Supplementary materials

Extended Method Section

Surgical induction of myocardial infarction in mice

Myocardial infarction was induced in 10–12 week old CXCR4-EGFP and C57BL/6 mice by surgical occlusion of the left anterior descending artery (LAD) through a left anterolateral approach. Mice were anesthetized by intraperitoneal injection of a mixture of 100 mg/kg ketamine (Sigma Chemical Co., 9, St. Louis, MO) and 5 mg/kg Xylazine (Sigma), intubated, and artificially ventilated by a mouse ventilator (HUGO SACHS, March, Germany) with 170 strokes/min and 200 µl/stroke. The chest cavity was opened by an incision of the left fourth intercostal space. The heart was exposed, the pericardial sac was opened and pulled apart, and the left anterior descending (LAD) artery was visualized. Ligation was proceeded with a 7-0 silk suture passed with a tapered needle underneath the LAD artery about 1-2 mm lower than the tip of the left auricle. Occlusion was confirmed by pallor of the anterior wall of the left ventricle. Lungs were overinflated, and the chest cavity, muscles, and skin were closed layer by layer with 4-0 silk sutures. After the operation, all mice received a single injection of analgesia with 10 mg/kg piritramid i.p. Afterwards the animals received further analgesia through drinking water (2.5 mg piritramid in 100 ml drinking water) for the next 7 days. Euthanasia at the end of the experiment was performed by cervical dislocation. Animal care and all experimental procedures were performed in strict accordance to the Austrian (BMWF-66.011/0031-II/3b/2014) and Directive 2010/63/EU of the European Parliament guidelines.

Fluorescent Activated Cell Sorting (FACS) of BM and heart cells

10-12 week old CXCR4-EGFP BAC transgenic reporter mice with or without LAD ligation were either treated with saline or DMOG (80 mg/kg/d), respectively, daily for up to 7 days. BM mononuclear cells were separated by density-gradient centrifugation using 1.077 g/ml

Histopaque solution (Sigma Chemicals), purified, and resuspended in PBS containing 1% BSA. Cells were incubated for 40 min in the dark at 4°C with the following fluoresceinisothiocyanate (FITC), phycoerythrin (PE), and peridininchlorophyll-protein (PerCP) conjugated monoclonal antibodies: CD45-PerCP, CD11b-PE, CD11b-PerCP, CD4-PE, CD20-PE, CD31-PE, CD34-PE, Flk-PE, CD86-PE, CD206-PE, F4/80-PE, CD133-PE, c-kit-PE, Sca-1-PE, CD3-biotin, CD45R/B220-biotin, CD11b-biotin, TER-119-biotin, and Ly-6G-biotin Abs (all from BD Pharmingen). Matching isotype antibodies (BD Pharmingen) served as controls. Cells were analysed by three-color flow cytometer using a Coulter Epics XL-MCLTM flow cytometer (Beckman Coulter). Each analysis included 50,000 events.

Cardiac cells from sham-operated and infarcted hearts of saline or DMOG treated CXCR4-EGFP reporter mice were analysed 2 days and 7 days after MI (n = 6), respectively. Therefore, a ‘‘myocyte-depleted’’ total heart cardiac cell suspension was prepared, incubating minced myocardium in 0.1% collagenase IV (GIBCO BrL) 30 min at 37°C. Cells were then filtered through a 70 mm mesh and stained with CD45-PerCP, CD11b-PE, CD11b-PerCP, CD4-PE, CD20-PE, CD31-PE, CD34-PE, Flk-PE, CD86-PE, CD206-PE, F4/80-PE, CD133-PE, c-kit-PE, Sca-1-PE, CD3-biotin, CD45R/B220-biotin, CD11b-biotin, TER-119-biotin, and Ly-6G-biotin Abs (all from BD Pharmingen). CXCR4-EGFP+ cells were analysed for co-expression of the previously mentioned cell markers using EPICS XLMCL flow cytometer and Expo32 ADC Xa software (Beckman Coulter). Each analysis included 50,000 events.

Expression analyses of SDF-1, CXCR4 and HIF-1 target genes in human HMEC-1, HAVSMC and murine BM cells

Human microvascular endothelial cells (HMEC-1) were purchased from CDC (Atlanta, GA) and grown in MCDB-131 medium (Gibco) containing 10% FCS (Life Technologies), 1% penicillin/streptomycin (Life Technologies), 1 µg/ml hydrocortisone (Sigma), 2 mM L-glutamine (Gibco), and 10 ng/ml EGF (Gibco). A line of human aortic vascular smooth muscle cells T/G HA-VSMC (VSMC; American Type Culture Collection, Manassas, VA)

established from a normal aorta was cultivated in Ham's F12K with L-glutamine (Gibco) containing 10 mM HEPES, 10 mM TES, 0.05 mg/ml ascorbic acid, 1% ITS (Life Technologies), 10% FSC (Life Technologies), 1% penicillin/streptomycin (Life Technologies), and 0.03 mg/ml endothelial cell growth supplement (Sigma). All cells were maintained at 37°C under humidified atmosphere of 5% $CO2$ and passaged with 0.25% trypsin/EDTA (Gibco) twice per week.

For gene expression analyses in BM cells, DMOG treated and control (C57BL/6J) mice were sacrificed by cervical dislocation and their femurs and tibiae were removed and cleaned. BM cells were obtained by flushing the bones with a syringe needle (27-gauge) in DMEM medium. Mononucleated BM cells were collected by a Ficoll density gradient centrifugation for 30 min (Histopaq 1083, Sigma). Cells were washed in PBS and dissolved in Trizol for the following RNA preparation. Total RNA was reversely transcribed using the QuantiTect RT kit (Qiagen) according to manufacturer´s protocol. Exon spanning primers for human and mouse SDF-1, CXCR4, VEGFA, PDK-1, LDHA, and ß-actin were designed and verified on agarose gels (Supplementary table 1). Using 2x SYBR green mastermix (Applied Biosystems, USA) quantitative gene expression was calculated using the comparative ΔΔCt-method.

Gene expression of HIF-1 target genes, fibrosis and hypertrophy genes in the heart

Heart tissues were minced and homogenized in Trizol reagent (Invitrogen, USA) and total RNA was isolated according to the manufacturer's instructions. RNA was reversely transcribed to cDNA using the QuantiTect RT kit (Qiagen). Exon spanning primers for mouse HIF-1α target genes VEGFA, LDHA, PDK-1, fibrosis marker collagen-1a and the hypertrophy marker BNP were designed and are listed in Supplementary Table 1. Using 2 x SYBR green mastermix (Applied Biosystems, USA) quantitative gene expressions was calculated using the comparative ΔΔCt-method with ß-actin as a reference gene.

Immunohistochemistry of CXCR4+ cells in the heart

In order to characterize SDF-1+ and CXCR4+ expressing cells in the ischemic heart, adult non-infarcted and adult infarcted hearts (day 7, day 30 after MI) were harvested, washed in phosphate-buffered saline, and immersion fixed in 4% paraformaldehyde for 24 hours at 4°C, cryoprotected in 30% sucrose, embedded in OTC (Sakura Finetek, Torrance, CA) and sectioned at 10 µm. Afterwards, CXCR4+ expressing cells were identified by EGFP immune reactivity and further characterized for co-expression of PECAM (endothelial cells; Sigma clone# 8A9) and CD11b (Alexa Fluor® 594 anti-mouse/human CD11b, Biolegend).

Functional parameters

To evaluate functional parameters, mice were randomly assigned to the following groups: Sham operated control mice (n = 5), saline-treated infarcted mice (n = 10), and DMOG (80 mg/kg i.p.) treated infarcted mice (n = 10). Pharmacological treatment was administered for up to 7 days post-MI (Supp. Fig. 4). Pressure-volume relations *in vivo* were analysed 30 days after MI. Mice were anesthetized with a mixture of 100 mg/kg ketamine (Sigma Chemical Co., 9, St. Louis, MO) and 5 mg/kg xylazine (Sigma), intubated, and ventilated (MiniVent, HUGO SACHS, Freiburg, Germany). Via the right carotid artery, an impedance micromanometer catheter (Millar Instruments, Houston, Texas) was introduced into the left ventricle. Raw conductance volumes were corrected for parallel conductance by the hypertonic saline dilution method as described previously [1]. Euthanasia at the end of the experiment was performed by cervical dislocation. Haemodynamic measurements and data analyses were performed independently by a blinded person using LabChart 8 analysis software (ADInstruments Ltd, United Kingdom).

Histology, immunostaining and quantification of apoptosis and neovascularization

30 days after MI hearts (n=10) were excised and fixed in 4% phosphate-buffered formalin. Thereafter, hearts were cut transversally into 2 mm thick slices and embedded in paraffin. Sections 10 µm thick were cut and mounted on positively charged glass slides. Standard

histological procedures (hematoxylin and eosin and Masson’s trichrome) and immunostaining were performed. Capillaries were stained with antibodies against CD31 (goat anti-mouse, Santa Cruz – SC1506), AEC was used as chromogen. Apoptotic cells were detected using the TUNEL assay (DeadEnd™ Fluorometric TUNEL System, Promega). Sections were co-stained with DAPI to detect all cell nuclei. Digital photographs were taken at a magnification of 400x, and four random high-power fields (HPFs) from the infarct border zone of each heart sample (n = 6) were analysed utilizing NIH Image software. For quantification, the apoptotic index (AI) was calculated as percentage of TUNEL+ nuclei (green) to total nuclei DAPI (blue). Scar size was calculated as the average of three transverse sections sampled at 2 mm intervals from the apex to the base using the following formula developed by Pfeffer et al.[2]: Scar size (%) = [transverse scar perimeter (epicardial plus endocardial)/total transverse perimeter (epicardial plus endocardial)] × 100. Infarct wall thickness was measured in Masson’s trichrome stained sections by taking the average length of five segments along evenly spaced radii from the centre of the LV through the infarcted and non-infarcted LV wall [3].

Statistical analysis

Results were expressed as mean ± SD. Multiple group comparisons were performed by one-way analysis of variance (ANOVA) followed by the Bonferroni procedure for comparison of means. Comparisons between two groups were performed using the unpaired two sided Student’s t test. Data were considered statistically significant at a value of $p<0.05$.

Supplementary tables

Table S1. Primer sequences for real-time PCR (h=human, m=mouse).

Gene	Forward primer	Reverse primer
**hSDF-1**	5´ ATGCCCATGCCGATTCTT 3´	5´ CACACTTGTCTGTTGTTGTTCTT 3´
**hCXCR4**	5´ TGTCATCACGCTTCCCTTC 3´	5´ GCTGTAGAGGTTGACTGTGTAG 3´
**hACTB**	5´ GCCGATCCACACGGAGTACT 3´	5´ CTGGCACCCAGCACAATG 3´
**hVEGFA**	5´ ATCAAACCTCACCAAGGCCA 3´	5´ CGGGATTTCTTGCGCTTTCG 3´
**hLDHA**	5´ GGCCTGTGCCATCAGTATCT 3´	5´ GATATCCACTTTGCCAGAGACA 3´
**hPDK-1**	5´ CCGCTCTCCATGAAGCAGTT 3´	5´ TGAACGGATGGTGTCCTGAG 3´
**mSDF-1**	5´ CATCAGTGACGGTAAACCAG 3´	5´ GCACAGTTTGGAGTGTTGAG 3´
**mCXCR4**	5´ CGGCTGTAGAGCGAGTGTTG 3´	5´ GCAGGGTTCCTTGTTGGAGT 3´
**mACTB**	5´ CGCCACCAGTTCGCCATGGA 3´	5´ TACAGCCCGGGGAGCATCGT 3´
**mVEGFA**	5´ GCACTGGACCCTGGCTTTAC 3´	5´ GTCTCAATCGGACGGCAGTA 3´
**mLDHA**	5´ TCCAGCAAAGACTACTGTG 3´	5´ GTTGGGAATGATGAACTTG 3´
**mPDK-1**	5´ TGCAAAGTTGGTATATCCAAAGCC 3´	5´ ACCCCGAAGCTCTCCTTGTA 3´
**mBNP**	5´ CCAGAGCAATTCAAGATGCAG 3´	5´ GGTCTTCCTACAACAACTTCAG 3´
**mCollagen-1a**	5´ GCTCCTCTTAGGGGCCACT 3´	5´ CCACGTCTCACCATTGGGG 3´

Supplementary figures

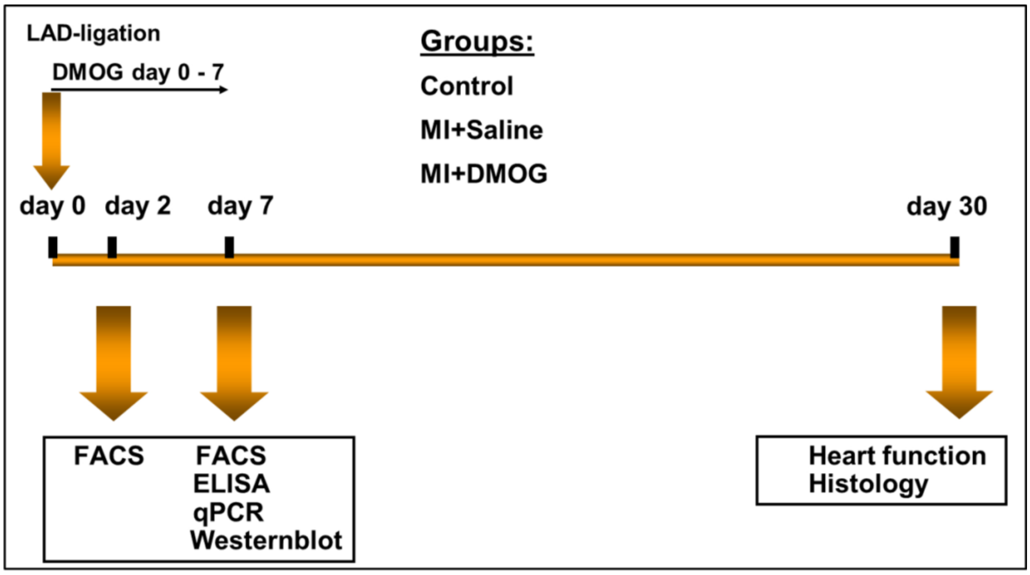

Fig. S1: Schematic representation of experimental design. To analyze gene and protein expression, C57BL/6 mice were sacrificed at day 7 after MI and ELISA, qPCR, and Western Blot analyses were performed. Cell recruitment was analyzed by FACS utilizing transgenic CXCR4-EGFP+ mice (n=10) at day 2 and 7 after MI. To evaluate myocardial function and histology, mice were randomly assigned to the following groups: Sham operated control mice (n = 5), saline-treated infarcted mice (n = 10), and DMOG (80 mg/kg i.p.) treated infarcted mice (n = 10). Immediately after LAD ligation, DMOG (80 mg/kg/d) was applied intraperitoneally (i.p.) for up to 7 days. 30 days after MI mice were analyzed with microcatheters and histology was performed.

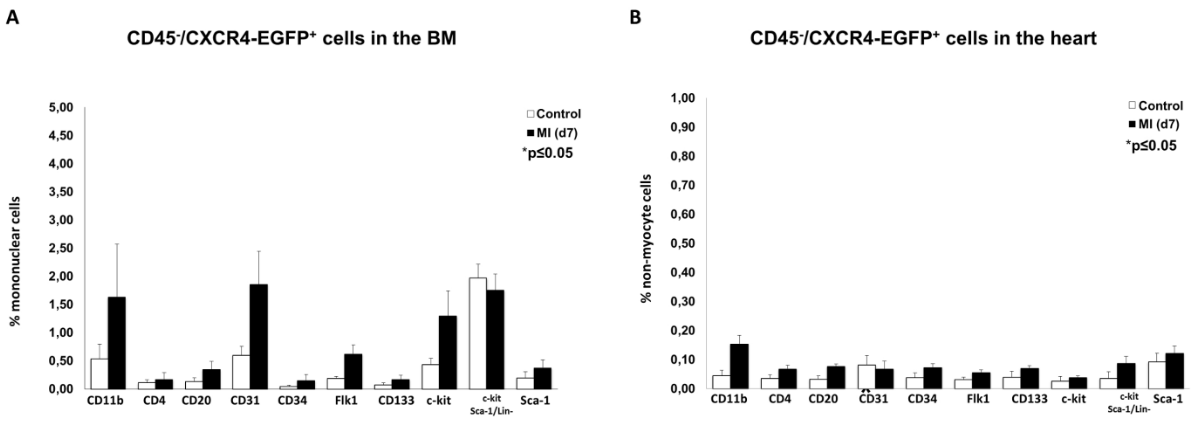

Fig. S2: FACS analyses of CXCR4-EGFP reporter mice for $CD45^-/CXCR4^+$ cells in the BM and ischemic heart. (A) Histograms of $CD45^-$/CXCR4-$EGFP^+$ cells in BM at day 7 ± MI. (B) Histograms of $CD45^-$/CXCR4-$EGFP^+$ cells in the heart at day 7 ± MI. All data represent mean ± SD (n = 4); Control vs. MI.

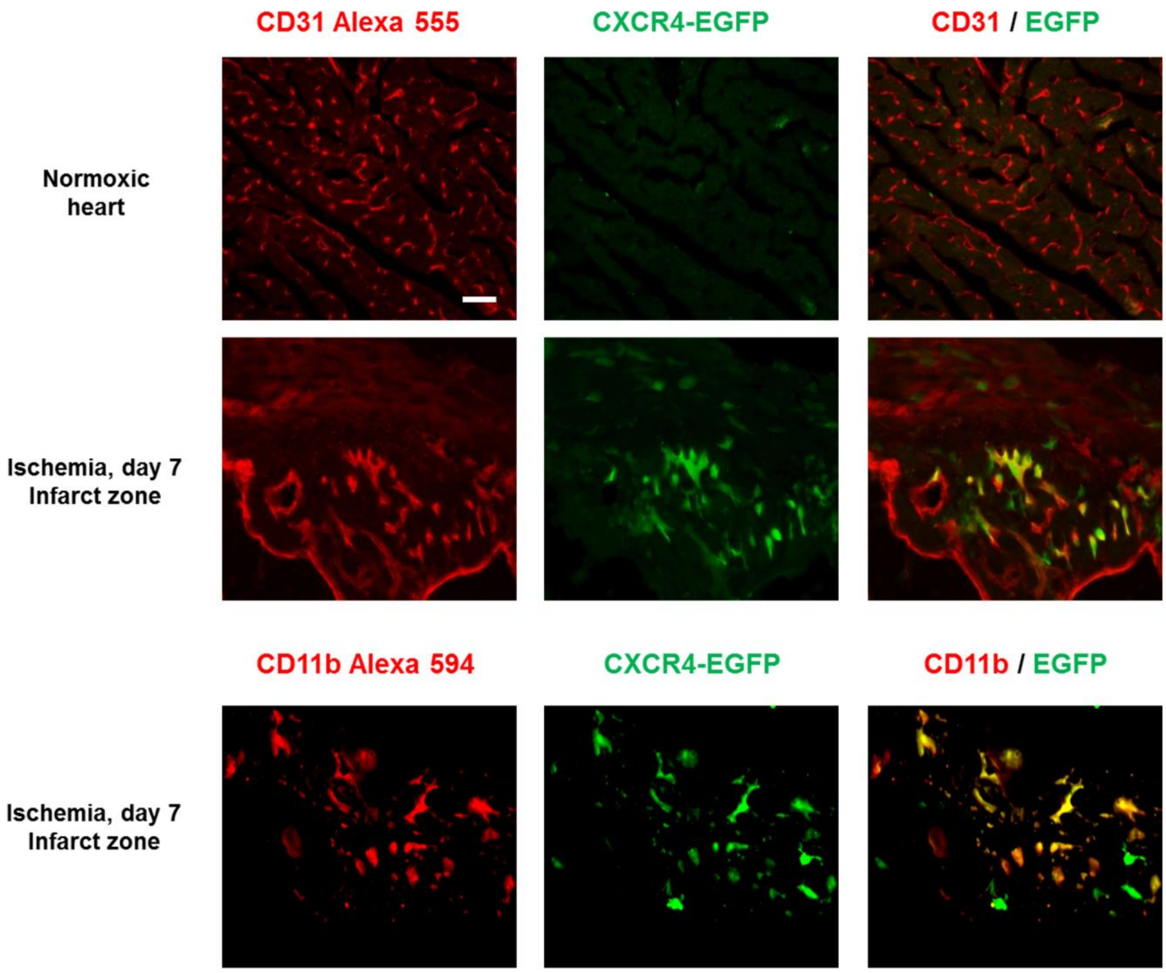

Fig. S3: CXCR4+ cells co-stained for CD31 and CD11b cells in the ischemic heart. In the normoxic heart CXCR4-EGFP+ cells were almost absent (upper row). Immunofluorescence images 7 days after ischemia in the infarcted area revealed substantial upregulation of CXCR4-EGFP+ cells in the heart. Co-staining of CXCR4-EGFP+ cells with the endothelial cell marker CD31 and the monocyte marker CD11b revealed co-expression in the infarct region (2nd row, 3rd row). Scale bar representing 25µm.

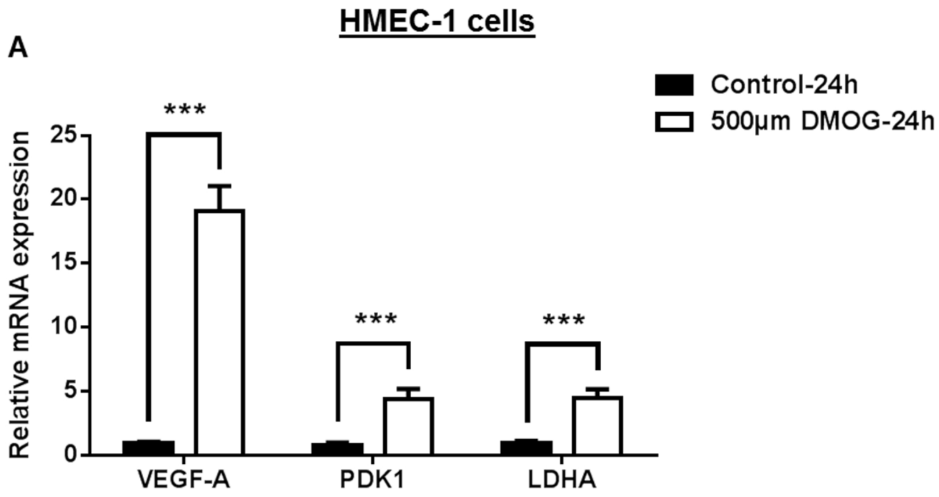

Fig. S4: DMOG activates HIF-1α target genes VEGFA, PDK-1, PDHA. HMEC-1 cells treated with 500 µM of DMOG for 24 h show an increase in mRNA levels of VEGF-A, PDK-1, and LDHA. Data represent mean ± SD (n = 6); ***P<0.001 Control vs. DMOG.

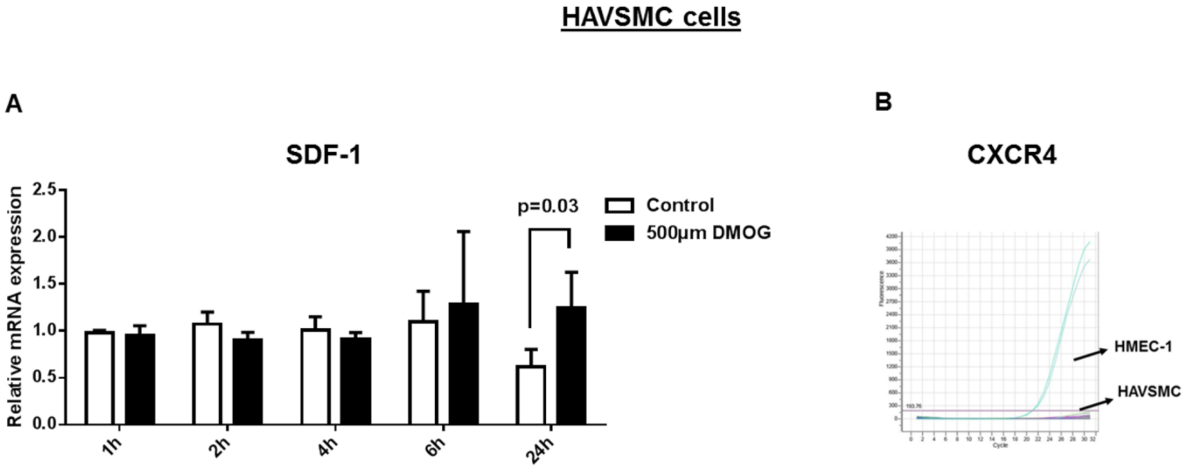

Fig. S5: DMOG increases SDF-1 expression in human aortic smooth muscle HAVSMC cells in time dependent manner. (A) SDF-1 mRNA levels in HAVSMC cells *in vitro* treated with 500 µM of DMOG for 1 h, 2 h, 4 h, 6 h and 24 h. Data represent mean ± SD (n = 6); Control vs. DMOG. (B) No CXCR4 amplification by qPCR analysis in HAVSMC cells.

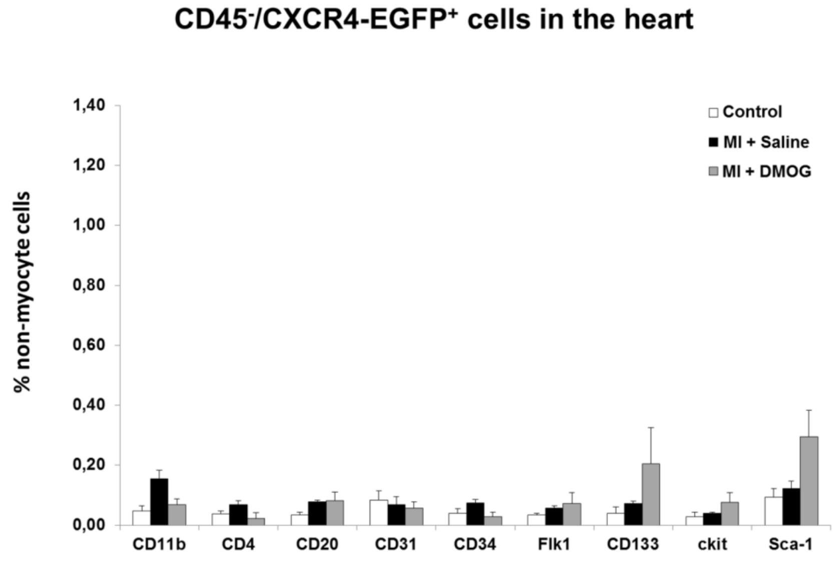

Fig. S6: DMOG has no effect on CD45⁻/CXCR4-EGFP⁺ cells in the ischemic heart. FACS analyses of CD45⁻/CXCR4-EGFP⁺ cells showed no increase in numbers of cells in the ischemic heart after DMOG treatment. All data represent mean ± SD (n = 4); Control vs. MI.

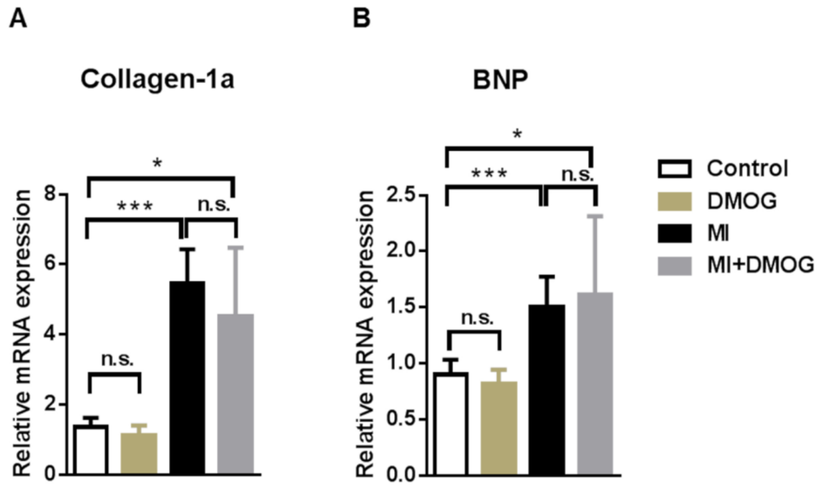

Fig. S7: Gene expression of fibrosis and hypertrophy genes in the heart. Expression of the fibrosis marker collagen-1a and the hypertrophy marker BNP in non-infarcted and infarcted hearts ± DMOG for 7 days. All data represent mean ± SD (n = 3); *P<0.05; ***P<0.001, n.s. not significant.

References to supplementary materials:

1. Zaruba MM, Huber BC, Brunner S, Deindl E, David R, Fischer R, Assmann G, Herbach N, Grundmann S, Wanke R, et al. (2008) Parathyroid hormone treatment after myocardial infarction promotes cardiac repair by enhanced neovascularization and cell survival. Cardiovasc Res 77: 722-731
2. Pfeffer JM, Pfeffer MA, Fletcher PJ, Braunwald E (1991) Progressive ventricular remodeling in rat with myocardial infarction. Am J Physiol 260: H1406-1414
3. Deindl E, Zaruba MM, Brunner S, Huber B, Mehl U, Assmann G, Hoefer IE, Mueller-Hoecker J, Franz WM (2006) G-CSF administration after myocardial infarction in mice attenuates late ischemic cardiomyopathy by enhanced arteriogenesis. Faseb J 20: 956-958
